# Risk factors for the histologic discrepancy of gastric adenomatous lesions and long-term outcome

**DOI:** 10.1097/MD.0000000000027827

**Published:** 2021-11-12

**Authors:** Jong Seok Joo, Jae Ho Park, Ju Seok Kim, Sun Hyung Kang, Hee Seok Moon, Jae Kyu Sung, Hyun Yong Jeong

**Affiliations:** Departments of Internal Medicine, Chungnam National University School of Medicine, Daejeon, Korea.

**Keywords:** endoscopic forceps biopsy, endoscopic resection, gastric adenoma

## Abstract

Although endoscopic forceps biopsies (EFB) have a significant role in diagnosing gastric adenoma, there are still discrepancies between EFBs and finalized pathology results.

Therefore, the objective of this study was to find the risk factors that cause this discrepancy and to analyze the effects of this discrepancy on the long-term outcome.

In this study patients that had received endoscopic resection due to low-grade gastric adenoma diagnosis from EFB between January of 2011 and January of 2018 at the Chungnam National University Hospital were retrospectively analyzed. According to whether there was histological discrepancy the cumulative incidence of the metachronous lesions were analyzed.

A total of 745 lesions diagnosed as low-grade gastric adenoma at EFB were enrolled, and the final pathology results were confirmed to be non-neoplastic (n = 19), low-grade adenoma (n = 614), High-grade adenoma (n = 63), and carcinoma (n = 49), and with the exception of non-neoplastic lesion, the results confirmed 84.6% (n = 614) for the concordant group and 15.4% (n = 112) for the discordant. The results of the multivariate analysis confirmed that depressed lesion (odds ratio [OR]: 2.056; 95% confidence interval [CI]: 1.130–3.451; *P* = .011), erythema (OR: 2.546; 95% CI: 1.604–4.030; *P* = .004), and a size >1.5 cm (OR: 1.903; 95% CI: 1.102–3.172; *P* = .018) were risk factors for discrepancy. The results also confirmed that for the average observation period of (SD) 39.12 (12.31) months, the cumulative incidence of metachronous neoplasm had a higher significance (*P* = 0.001) in the discordant group when compared to that of the concordant group.

The factors related to the histologic discrepancy of low-grade gastric adenoma were depressed lesion, erythema and size >1.5 cm. In the groups with histological discrepancy, the cumulative incidence of the metachronous neoplasm was significantly higher and therefore closer observation of such patients after performing endoscopic resection is necessary.

## 1 Introduction

Gastric adenomas are known to be precancerous lesions of gastric cancer, and endoscopic forceps biopsy (EFB) is considered to be the gold standard method for performing histological diagnosis of gastric adenoma.^[1,2]^ But because EFB diagnoses are performed by extracted only a partial sample of the lesion, because the entire tissue is not diagnosed, there are histological discrepancies that occur when compared to the final pathology results of endoscopic resection patients.^[3]^ Due to these discrepancies, high-grade adenomas or carcinoma can be underestimated as low-grade adenomas, which can lead to negative development in the clinical outcome of patients due to improper treatment. Therefore the accurate diagnosis of gastric neoplastic lesions is extremely important and many research studies are being conducted to increase the accuracy of this diagnosis.

Recently if efficacy is possible for the treatment of gastric neoplastic lesion including early gastric cancer, endoscopic resections, including endoscopic mucosal resections (EMR) and endoscopic submucosal dissection (ESD), are the main type of procedure that are performed.^[4,5]^ These types of endoscopic resection, when compared to surgery, are able to preserve the stomach and are beneficial in the quality of life of the patients, and according to recent research, these procedures have a high curative resection rate and also have favorable long-term outcome.^[6,7]^ Therefore if accurate diagnosis is possible through EFB, the results of treating gastric neoplastic lesion through endoscopic resection is on the favorable side. Although there are many related factors to the histologic discrepancy of gastric adenoma including the lesion size, presence of erythema or nodularity, depressed lesion, erosion or ulcer, among others, the results of various research studies have been shown to be inconsistent.^[8–10]^ There was also a lack of research on the effects of this histological discrepancy on the long-term outcome of patients.

Therefore the objectives of this research were to analyze patients diagnosed with low-grade gastric adenoma using EFB, and to discover the frequency of histological discrepancy that overs and the risk factors related to this discrepancy. The effects of histological discrepancy on the long-term outcome of patients, including patients with metachronous lesions, were also examined.

## 2 Methods

### 2.1 Patients

The medical records of patients at the Chungnam National University Hospital (Daejeon, Korea) that had endoscopic resection performed due to low-grade gastric adenoma diagnosis from EFB between the periods of January 2011 to January of 2018 were retrospectively analyzed. The research subject of this study were patients older than 18 years of age that had been continuously followed up with for longer than a duration of 1 year after the endoscopic resection was performed, and with the exception of cases where non-neoplastic lesions were confirmed in the pathology results of the endoscopic resections, these patient cases were categorized into low-grade adenoma, high-grade adenoma, and carcinoma. The pathology results for the pathology results after the endoscopic resections had been performed were categorized according to the World Health Organization classification of pathology. For the location of the lesions, the stomach was divided equally into 3 parts in including the upper, middle, and lower third. The gross type and size of the lesion from a morphological basis was also confirmed, and whether to not there was intestinal metaplasia or atrophy and presence of *H pylori* infections were also confirmed. This study was conducted retrospectively through medical chart review and was not approved by the institutional review board.

### 2.2 Endoscopic resection procedure

All patients enrolled in the study received endoscopic resections, which included EMR and ESD procedures, from expert endoscopists working at the Chungnam National University Hospital. The procedure was performed using white-light endoscopy, and indigo-carmine solution was applied or narrow-band imaging was used to clearly mark the boundaries of the lesion. The endoscopic resection were performed according to previously well-known methods and the final decisions for whether to perform EMR or ESD were made by the gastrointestinal (GI) endoscopist according to the location and size and shape of the lesions. High frequency generators (ICC200 or VIO 300D; ERBE Elekromedizin, Tubingen, Germany) were used to perform the endoscopic resection. The follow-up examinations including chest radiography and a complete blood cell count were recorded to monitor the patient for complications. Through monitored examination of the patient, including performing endoscopy and abdominal computed tomography after the endoscopic resections were performed, according to the final pathology results the appropriate method and frequency for patient examination were determined by the doctor.

### 2.3 Histologic results and discrepancy

All pathology slides were evaluated by the GI pathologist at our hospital. Because frequency and excessive tissue examination can cause ulcers of submucosal fibrosis, causing difficulties with the desquamation during endoscopic resections, EFBs were performed outside of the hospital and when the patient was transferred to the hospital either EFBs were not additionally performed or only performed as necessary. But the tissue slides generated at external hospitals were reviewed by our hospital pathologist. The cut tissue was fixed to the polystyrene plate using a pin, and after fixating in 10% formalin, serial sections were taken at 2 mm intervals and were examined using a microscope. When various different tissue cells were mixed in together, the tissue cells that made up >50% of the entire tissue were categorized as the main tissue type.

According to the final pathology results were categorized as non-neoplastic, low-grade adenoma, high-grade adenoma, and carcinoma. Excluding the non-neoplastic lesions, low-grade adenomas were categorized as the concordant group, and high-grade adenoma and carcinoma were categorized as the discordant group. By comparing the baseline characteristics and endoscopic resection results of the 2 groups, the risk factors for histological discrepancy to occur were analyzed. Also by confirming the cumulative incidence and survival of the metachronous neoplasm for the concordant and discordant groups to analyze the effect that histologic discrepancy had on the long-term outcome of the patients. Metachronous neoplasm was defined as gastric neoplasm that was discovered 1 year after endoscopic resections had been performed.

### 2.4 Statistical analysis

To compare the categorical variables including baseline patients characteristics, endoscopic findings and endoscopic resection results, and so on, *χ*^2^ testing and Fisher exact testing were performed. The categorical variables were indicated as frequencies and percentage and continuous variables were indicating as means and standard deviation (SD). For multivariate analysis, the factors confirmed to have a *P* value of <.1 in the univariate analysis were adjusted to analyze using a logistic regression model and indicated as odds ratio (OR) and confidence interval (CI). The Kaplan-Meier method was used to confirm the cumulative incidence of the metachronous neoplasm for the concordant and discordant groups, and the 2 groups were compared using the log-rank test. Significant was defined as when the *P* values were 2-sided and below the value .05. All statistical analyses were conducted using SPSS version 19.0 (SPSS Inc., Chicago, IL).

## 3 Results

### 3.1 Patient characteristics

**Figure 1:**
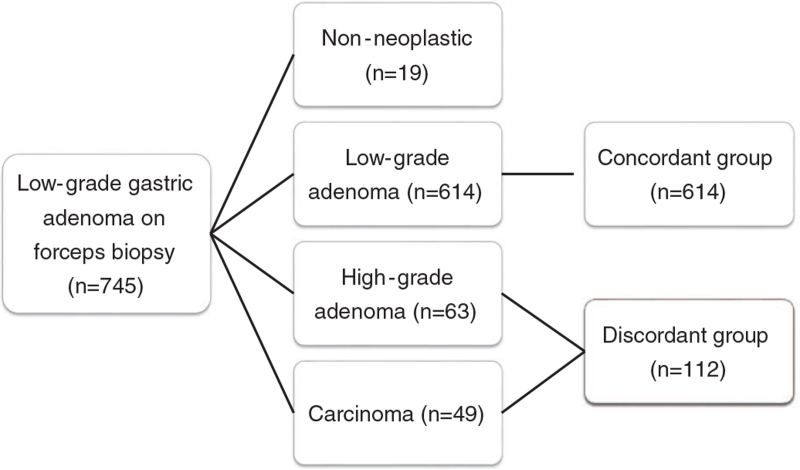
Flow chart of patients.

A total of 745 lesions were enrolled from the 713 patients diagnosed with low-grade adenoma diagnosed using EFB. The final pathology results after having endoscopic resections performed were confirmed to be non-neoplastic 2.6% (n = 19), low-grade adenoma 82.4% (n = 614), high-grade adenoma 8.5% (n = 63), and carcinoma 6.5% (n = 49) (Fig. 1). With the exception of non-neoplastic lesions, the concordant group was 84.6% (n = 614), and the discordant group was 15.4% (n = 112). The overall histologic discrepancy rate of the low-grade gastric adenoma including the non-neoplastic lesion was confirmed to be 17.6% (131/745), and when excluding non-neoplastic lesion the results were confirmed to be 15.4% (112/726).

The basic characteristics of the 726 patients are summarized as shown in Table 1. The mean age (SD) was confirmed to be 63.42 (8.76) year of age with 67.9% (n = 493) of patients being male. From a morphologically, macroscopic elevated type was the most prevalent type at 63.6% with most of the lesions occurring at the lower third (64.1%) location. Intestinal metaplasia was discovered in 49.7% (n = 361) of patients and 60.3% (n = 438) of patients were discovered to have *H pylori* infection. EMR (72.7%) was the more frequent endoscopic resection method that was used and the results confirmed that the average size of the lesion was (SD) 1.87 (0.77) cm and the average frequency for EFBs being performed was 2.01 (0.98).

**Table 1:** Baseline characteristics.

Characteristics, no. (%)	Total patients (n = 726)
Age, y (mean, SD)	63.42 (8.76)
Sex (male)	493 (67.9)
Gross type	
Elevated	462 (63.6)
Flat	151 (20.8)
Depressed	113 (15.6)
Location of lesion	
Upper third	30 (4.1)
Middle third	231 (31.8)
Lower third	465 (64.1)
Intestinal metaplasia	361 (49.7)
*H pylori* infection	438 (60.3)
Method of endoscopic resection	
EMR	528 (72.7)
ESD	198 (27.3)
Lesion diameter (SD), cm	1.87 (0.77)
Number of biopsy (SD)	2.01 (0.98)

EMR = endoscopic mucosal resection, ESD = endoscopic submucosal dissection, *H pylori* = *Helicobacter pylori*, SD = standard deviation.

### 3.2 Comparison of concordant and discordant group

After performing endoscopic resection, the clinical characteristics confirmed in the concordant and discordant group can be summarized as follow (Table 2). The average age for the concordant group (n = 614) was confirmed to be (SD) 62.93 (9.04) years old with 68.9% (n = 423) male patients, and the average age for the discordant group (n = 112) was confirmed to be (SD) 64.27 (10.34) year's old, with 62.5% (n = 70) male patients, confirming that there were no significant differences between the 2 groups. Additionally there were no significant differences between the 2 groups in terms of location of lesion, whether or not patients had *H pylori* infection, endoscopic resection method, lesion size, frequency of EFB, and presence of erosion and atrophy. The discordant group when compared to the concordant group, the number of depressed type was significantly higher (25.0% vs 13.8%, *P* = .005). The 2 groups were also shown to have significant differences for intestinal metaplasia (*P* = .005) and erythema of the surface of the lesion (*P* = .000).

**Table 2:** Clinical characteristics of concordant and discordant group after endoscopic resection.

Characteristics no. (%)	Concordant (n = 614)	Discordant (n = 112)	*P*
Age, y (SD)	62.93 (9.04)	64.27 (10.34)	.349
Sex (male)	423 (68.9)	70 (62.5)	.222
Gross type			.104
Elevated	398 (64.8)	64 (57.1)	
Flat	131 (21.3)	20 (17.9)	
Depressed	85 (13.8)	28 (25.0)	
Location			.670
Upper third	23 (3.7)	7 (6.3)	
Middle third	192 (31.3)	39 (34.8)	
Lower third	399 (65.0)	66 (58.9)	
Intestinal metaplasia	291 (47.4)	70 (62.5)	.005
*H pylori* infection	376 (61.2)	68 (60.7)	1.000
Method of endoscopic resection			.652
EMR	449 (73.1)	79 (70.5)	
ESD	165 (26.9)	33 (29.5)	
Lesion diameter, cm			.471
<1.0	194 (31.6)	29 (25.9)	
≥1.0 and <2.0	306 (49.8)	57 (50.9)	
≥2.0	114 (18.6)	26 (23.2)	
No. of biopsy (SD)	1.93 (1.17)	2.20 (1.42)	.203
Erythema	128 (20.8)	46 (41.1)	.000
Erosion	104 (16.9)	27 (24.1)	.092
Atrophy	387 (63.0)	69 (61.6)	.857

EMR = endoscopic mucosal resection, ESD = endoscopic submucosal dissection, *H pylori* = *Helicobacter pylori*, SD = standard deviation.

### 3.3 Risk factors for histologic discrepancy

To discover the risk factors for histologic discrepancy by adjusting the depressed lesion, intestinal metaplasia, erosion, erythema and lesion diameter, multivariate analysis was performed (Table 3). The results confirmed that depressed lesion (OR: 2.056; 95% CI: 1.130–3.451; *P* = .011), erythema (OR: 2.546; 95% CI: 1.604–4.030; *P* = .004), and size >1.5 cm (OR: 1.903; 95% CI: 1.102–3.172; *P* = .018) were factors that were related to histologic discrepancy of gastric low-grade adenoma. However the results did not confirm any statistically significant differences between the 2 groups for intestinal metaplasia (OR: 1.695; 95% CI: 0.972–2.707; *P* = .078) and erosion (OR: 1.458; 95% CI: 0.875–2.519; *P* = .108).

**Table 3:** Multivariate analysis of risk factors for histologic discrepancy between forceps biopsy and endoscopic resection.

Variables	OR (95% CI)	*P*
Depressed lesion	2.056 (1.130–3.451)	.011
Intestinal metaplasia	1.695 (0.972–2.707)	.078
Erosion	1.458 (0.875–2.519)	.108
Erythema	2.546 (1.604–4.030)	.004
Lesion diameter, cm		.018
<1.5	1 (Reference)	
≥1.5	1.903 (1.102–3.172)	

CI = confidence interval, OR = odds ratio.

### 3.4 Long-term outcome

**Figure 2:**
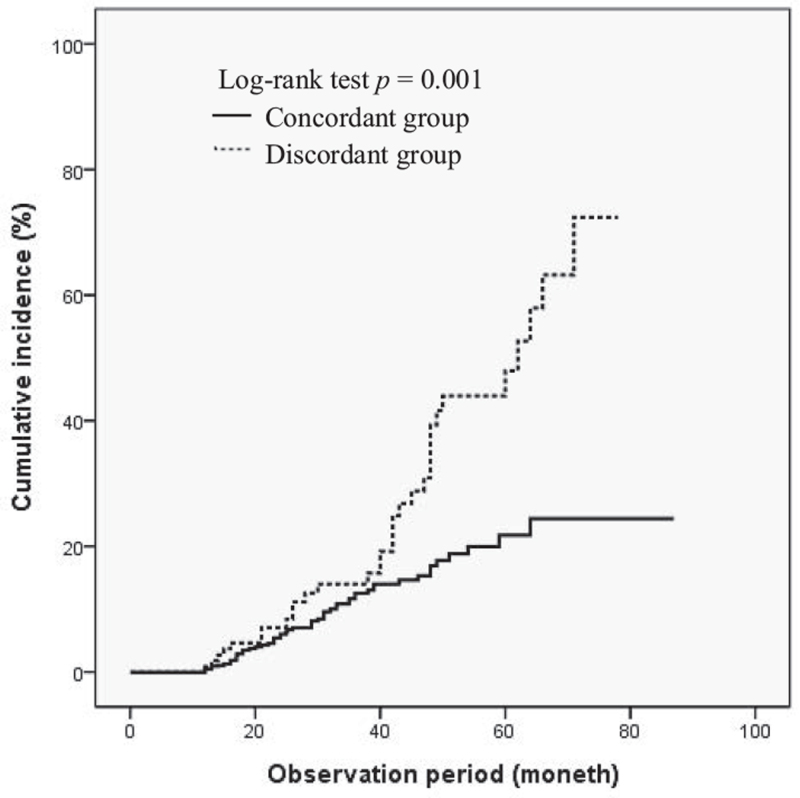
Cumulative incidence of metachronous neoplasm.

To examine the effects that histologic discrepancy that occurs in gastric adenoma have on the long-term outcome of patients, the cumulative incidence of metachronous neoplasm for both the concordant and discordant groups was analyzed using the Kaplan-Meier method (Fig. 2). The results confirmed that the metachronous lesions newly discovered from the total number of research subjects during the average (SD) 39.12 (12.31) months of observation were a total of 88lesions (concordant group (n = 56) and discordant group (n = 32)). The concordant group was observed for an average of (SD) 38.91 (13.84) months and the discordant group was observed for an average of (SD) 41.02 (16.13) months, with no statistically significant difference between 2 groups (*P* = .595). Most of the metachronous lesions were treated using endoscopic resections and the three patients discovered to have invasive carcinoma surgical resections were performed and have been follow-up to not recurrence. There were no deaths caused by gastric lesions in either of the groups during the period of observation. The log-rank test confirmed that when compared to the concordant group, the discordant group was confirmed to have a significantly higher cumulative incidence of metachronous lesions (*P* = .001). The 5-year cumulative incidence of metachronous lesions was confirmed to be 22.8% for the concordant group and 43.8% for the discordant group.

## 4 Discussion

Although the results of this research confirmed low-grade gastric adenoma in EFB, the final pathology results after the endoscopic resection was performed was confirmed to show an overall histologic discrepancy rate of 17.6% (131/745), which is similar to that of the results of previous research (17.4%–44.5%).^[11,12]^ The reason for this histologic discrepancy is because it is not easy to histologically distinguish between low or high-grade adenoma, carcinoma using small endoscopic biopsy specimen.^[13]^ Also because only partial sample of lesions are extracted when conducting EFB, which are unable to represent the entire tissue, when there are cases of focal high-grade adenoma within low-grade adenoma and sampling errors occur.^[14]^ There are many research studies being conducted to minimize this type of histologic discrepancy and the narrow-band imaging (NBI) endoscopy method, which is used as a replacement for the previous white endoscopy is one of these methods.^[15]^ There are research studies that claim to have observed that NBI endoscopy has shown 95% sensitivity and 96% specificity for the diagnosis of gastric neoplasm, but there are also research studies that report opposite findings and therefore there is controversy in applying these findings clinically and there is a need for further research on this issue.^[16,17]^

When these histologic discrepancies, which can occur due to limitations of EFB, lead to inappropriate treatments due to underestimating high-grade adenoma or carcinoma as low-grade adenoma, this will have a negative effect on the long-term outcome for the patient. However, there is still a lack of the risk factors that are related to histologic discrepancy.^[8–10]^ According to one research study, surface redness (OR: 6.493; 95% CI: 2.557–16.666; *P* = .001), nodularity (OR: 2.762; 95% CI: 1.237–6.172; *P* = .013), and size l>1 cm (OR: 3.496; 95% CI: 1.375–8.849; *P* = .009) were risk factors for histologic discrepancy in low-grade dysplasia lesions.^[18]^ This research confirmed similar results showing that depressed lesion (OR: 2.056; 95% CI: 1.130–3.451; *P* = .011), erythema (OR: 2.546; 95% CI: 1.604–4.030; *P* = .004), and the size >1.5 cm (OR: 1.903; 95% CI: 1.102–3.172; *P* = .018) were risk factors for histologic discrepancy. In summary when these risk factors are present, there is a need for more in-depth examination and monitoring to reduce histologic discrepancy, which is closely related to the long-term outcome of patients.

In the case of high-grade adenoma or carcinoma, treatment such as an endoscopic resection or surgery is necessary but, there is controversy whether this treatment is necessary in low-grade adenoma.^[19,20]^ The Vienna classification recommends that endoscopic resections should be performed for noninvasive low-grade neoplasm or for follow-up procedures to be taken.^[21]^ Because there are cases where regression naturally occurs for low-grade adenoma and its malignancy potency is low, there are claims that endoscopic resections are not necessary but there are research studies that claim opposing results.^[22,23]^ It is well known that low-grade adenoma is a precancerous lesion, and because pathology results can change after endoscopic resections including EFBs have been performed, only performing follow-up monitoring for patients when low-grade adenoma was diagnosed by EFB is risky. Also due to recent developments and advancements in endoscopic techniques, the procedure-related complication is low and favorable clinical outcomes have been shown, endoscopic resections are widely used to conduct both accurate diagnoses and treatments at the same time.^[6,7]^ This research study also supports these claims based on histologic discrepancies being discovered in 17.6% of patients diagnosed with low-grade adenoma using EFP.

In this study, the cumulative incidence of the metachronous lesion was significantly higher in the discordant group when compared to the concordant group, and therefore it could be confirmed that histologic discrepancy had an effect on the long-term outcome of patients. And there were no patient deaths related to gastric lesion during the observation period. The fact that histologic discrepancy is closely related to the long-term outcome of the patient can be said to be additional evidence that shows the risks associated with only following up with patients diagnosed with low-grade adenoma.

The limitations of this study are as follows. First, because this was a retrospective and single-center study, it is difficult to generalize the findings but because the number of patients enrolled in the study is larger in comparison to other studies, the limitations related to this are considered to be not significant. Also although the relationship between the cumulative incidence of the metachronous lesion and the histologic discrepancy was confirmed, there is a lack of analysis on why these results occurred. However, this study is significant in that it analyzed the relationship between histologic discrepancy and the incidence of metachronous lesions, which is an area that has not been previously researched in depth.

The histologic discrepancy of low-grade adenoma was confirmed in 17.6% of patients, and depressed lesion, erythema, and lesion size >1.5 cm were risk factors for histologic discrepancy. Also histologic discrepancy increased the cumulative incidence of metachronous lesions, this had a negative effect on the long-term outcome of patients. In conclusion, endoscopist should be aware of the possibility of histologic discrepancy during endoscopic resection, and always more detailed lesion observation is required.

## Author contributions

**Conceptualization:** Ju Seok Kim.

**Data curation:** Jong Seok Joo, Ju Seok Kim, Sun Hyung Kang.

**Formal analysis:** Jong Seok Joo, Jae Ho Park.

**Funding acquisition:** Ju Seok Kim.

**Investigation:** Jong Seok Joo, Ju Seok Kim, Hyun Yong Jeong.

**Project administration:** Ju Seok Kim, Sun Hyung Kang, Hee Seok Moon, Jae Kyu Sung.

**Resources:** Jong Seok Joo.

**Software:** Hyun Yong Jeong.

**Supervision:** Jae Kyu Sung, Hyun Yong Jeong.

**Validation:** Hee Seok Moon.

**Visualization:** Jae Kyu Sung, Hyun Yong Jeong.

**Writing – original draft:** Jae Ho Park.

**Writing – review & editing:** Ju Seok Kim.

## References

[1] Ming SC Bajtai A Correa P. Gastric dysplasia. Significance and pathologic criteria. Cancer 1984;54:1794–801. 6478415 10.1002/1097-0142(19841101)54:9<1794::aid-cncr2820540907>3.0.co;2-w

[2] Maekawa A Kato M Nakamura T. Incidence of gastric adenocarcinoma among lesions diagnosed as low-grade adenoma/dysplasia on endoscopic biopsy: a multicenter, prospective, observational study. Dig Endosc 2018;30:228–35. 29094455 10.1111/den.12980

[3] Weinstein WM Goldstein NS. Gastric dysplasia and its management. Gastroenterology 1994;107:1543–5. 7605417 10.1016/0016-5085(94)90561-4

[4] Oka S Tanaka S Kaneko I. Advantage of endoscopic submucosal dissection compared with EMR for early gastric cancer. Gastrointest Endosc 2006;64:877–83. 17140890 10.1016/j.gie.2006.03.932

[5] Tanaka M Ono H Hasuike N. Endoscopic submucosal dissection of early gastric cancer. Digestion 2008;77:23–8. 18204258 10.1159/000111484

[6] Chung IK Lee JH Lee SH. Therapeutic outcomes in 1000 cases of endoscopic submucosal dissection for early gastric neoplasms: Korean ESD Study Group multicenter study. Gastrointest Endosc 2009;69:1228–35. 19249769 10.1016/j.gie.2008.09.027

[7] Choi MK Kim GH Park DY. Long-term outcomes of endoscopic submucosal dissection for early gastric cancer: a single-center experience. Surg Endosc 2013;27:4250–8. 23765426 10.1007/s00464-013-3030-4

[8] Cho SJ Choi IJ Kim CG. Risk of high-grade dysplasia or carcinoma in gastric biopsy-proven low-grade dysplasia: an analysis using the Vienna classification. Endoscopy 2011;43:465–71. 21425043 10.1055/s-0030-1256236

[9] Lee CK Chung IK Lee SH. Is endoscopic forceps biopsy enough for a definitive diagnosis of gastric epithelial neoplasia? J Gastroenterol Hepatol 2010;25:1507–13. 20796147 10.1111/j.1440-1746.2010.006367.x

[10] Lim H Jung HY Park YS. Discrepancy between endoscopic forceps biopsy and endoscopic resection in gastric epithelial neoplasia. Surg Endosc 2014;28:1256–62. 24310738 10.1007/s00464-013-3316-6

[11] Correa P Haenszel W Cuello C. Gastric precancerous process in a high risk population: cross-sectional studies. Cancer Res 1990;50:4731–6. 2369747

[12] Sung HY Cheung DY Cho SH. Polyps in the gastrointestinal tract: discrepancy between endoscopic forceps biopsies and resected specimens. Eur J Gastroenterol Hepatol 2009;21:190–5. 19092673 10.1097/MEG.0b013e3283140ebd

[13] Kato M Nishida T Tsutsui S. Endoscopic submucosal dissection as a treatment for gastric noninvasive neoplasia: a multicenter study by Osaka University ESD Study Group. J Gastroenterol 2011;46:325–31. 21107615 10.1007/s00535-010-0350-1

[14] Zhao G Xue M Hu Y. How commonly is the diagnosis of gastric low grade dysplasia upgraded following endoscopic resection? A meta-analysis. PLoS One 2015;10:e0132699. 26182344 10.1371/journal.pone.0132699 PMC4504521

[15] Hirata I Nakagawa Y Ohkubo M. Usefulness of magnifying narrow-band imaging endoscopy for the diagnosis of gastric and colorectal lesions. Digestion 2012;85:74–9. 22269282 10.1159/000334642

[16] Yao K Iwashita A Tanabe H. White opaque substance within superficial elevated gastric neoplasia as visualized by magnification endoscopy with narrow-band imaging: a new optical sign for differentiating between adenoma and carcinoma. Gastrointest Endosc 2008;68:574–80. 18656862 10.1016/j.gie.2008.04.011

[17] Yao K Iwashita A Nambu M. Nature of white opaque substance in gastric epithelial neoplasia as visualized by magnifying endoscopy with narrow-band imaging. Dig Endosc 2012;24:419–25. 23078433 10.1111/j.1443-1661.2012.01314.x

[18] Choi CW Kim HW Shin DH. The risk factors for discrepancy after endoscopic submucosal dissection of gastric category 3 lesion (low grade dysplasia). Dig Dis Sci 2014;59:421–7. 24366779 10.1007/s10620-013-2874-8

[19] Hirota WK Zuckerman MJ Adler DG. Standards of Practice Committee, American Society for Gastrointestinal Endoscopy. ASGE guideline: the role of endoscopy in the surveillance of premalignant conditions of the upper GI tract. Gastrointest Endosc 2006;63:570–80. 16564854 10.1016/j.gie.2006.02.004

[20] Rugge M Cassaro M Di Mario F. Interdisciplinary Group on Gastric Epithelial Dysplasia (IGGED). The long term outcome of gastric non-invasive neoplasia. Gut 2003;52:1111–6. 12865267 10.1136/gut.52.8.1111 PMC1773761

[21] Schlemper RJ Riddell RH Kato Y. The Vienna classification of gastrointestinal epithelial neoplasia. Gut 2000;47:251–5. 10896917 10.1136/gut.47.2.251 PMC1728018

[22] Yamada H Ikegami M Shimoda T. Long-term follow-up study of gastric adenoma/dysplasia. Endoscopy 2004;36:390–6. 15100945 10.1055/s-2004-814330

[23] Park SY Jeon SW Jung MK. Long-term follow-up study of gastric intraepithelial neoplasias: progression from low-grade dysplasia to invasive carcinoma. Eur J Gastroenterol Hepatol 2008;20:966–70. 18787462 10.1097/MEG.0b013e3283013d58

